# Supplementary material for: Content-rich biological network constructed by mining PubMed abstracts
Source: BMC Bioinformatics. 2004 Oct 8;5:147. doi: 10.1186/1471-2105-5-147 (PMC528731; doi:10.1186/1471-2105-5-147)
Supplement: Additional File 5 — The original Chilibot query results of the term "long-term potentiation (LTP)" and 22 other terms, limiting the latest references analyzed to the years 1990, 1995, 2000, and 2004. [file 1471-2105-5-147-S5.bz2 › chilibotAdditionalFile5/ltp2004/html/ERK_CREB.html]

 


 **ERK** and **CREB** 
  
Found 162 abstracts in PubMed,  **30 abstracts were retrieved and analyzed**.  


---

 Search Google  |
 PDF files only 
|  EDU domain only 

---

**Interactive relationship** (e.g. stimulation, inhibition, etc)

- However, activation of  **ERK**  did not involve in  **CREB**  phosphorylation or concomitantly StAR expression.  Ref: 12933667 Endocrinology, 2003
- We show that vGPCR induced activation of AP 1 and  **CREB**  is mediated cooperatively by a Gq  **ERK**  1 2 and a Gi PI3K Src axis.  Ref: 14724579 Oncogene, 2004
- **ERK**  activation by the MAPK  **ERK**  kinase MEK leading to cyclic AMP response element binding protein  **CREB**  phosphorylation is implicated in the formation of long term memory.  Ref: 12946695 Neuroscience, 2003
- These results indicate that lead activated PKC, CaMKII and MEK  **ERK**  p90RSK pathways simultaneously, all of which contributed to  **CREB**  phosphorylation.  Ref: 12832008 Brain Res Bull, 2003
- We conclude that lead activates PKC, CaMKII or MEK  **ERK**  p90RSK pathways in GT1 7 cells, leading to  **CREB**  phosphorylation and modulation of gene expression.  Ref: 12832008 Brain Res Bull, 2003
- In the hypothalamic arcuate nucleus, phosphorylation of  **ERK**  significantly increased during fasting, spatially coincident with phosphorylation of cAMP response element binding protein  **CREB** , induction of c Fos, and expression of neuropeptide Y NPY.  Ref: 14763996 J NeuroendocrinolJ Neuroendocrinol, 2003
- We report that, identically to 17beta estradiol or dihydrotestosterone, but differently from raloxifene, estren alters the activity of Elk 1, CCAAT enhancer binding protein beta C EBPbeta, and cyclic adenosine monophosphate response element binding protein  **CREB** , or c Jun c Fos by an extranuclear action of the ER or AR, resulting in activation of the Src Shc  **ERK**  pathway or downregulation of JNK, respectively.  Ref: 12782668 J Clin Invest, 2003

- :-)- Further, transfection of the HA FAK Y397F construct decreased FAK, MAPK  **ERK** , and  **CREB**  phosphorylation, and the inhibition of MAPK  **ERK**  decreased  **CREB**  phosphorylation.  Ref: 12764094 J Neurosci, 2003

  **Parallel relationship** (e.g. studied together, co-existance, homology, etc.)

  - In conclusion, IL 1 beta was found to induce MUC8 gene expression via a sequential  **ERK**  RSK1  **CREB**  pathway in human airway epithelial cells.  Ref: 12842905 J Biol Chem, 2003
  - Induction of MUC8 gene expression by interleukin 1 beta is mediated by a sequential  **ERK**  MAPK RSK1  **CREB**  cascade pathway in human airway epithelial cells.  Ref: 12842905 J Biol Chem, 2003
  - In the arcuate nucleus of ob ob mice, phosphorylations of  **ERK**  and  **CREB**  were decreased during fasting.  Ref: 14763996 J NeuroendocrinolJ Neuroendocrinol, 2003
  - Remarkably, caspase dependent cleavage of NF kappaB family members as well as of Akt and  **CREB**  proteins... became prominent at 24 h, a time point coincident with the peak of caspase dependent apoptosis.  Ref: 12807432 J Neurochem< MedlineTA>J Neurochem, 2003
  - Treatment with nonmyocyte conditioned media stimulated the phosphorylation of  **ERK** , Akt, and cAMP response element binding protein  **CREB**  in myocytes.  Ref: 14736733 EndocrinologyEndocrinology, 2003
  - Activation of EP2 receptor induced phosphorylation of  **ERK**  and cAMP response element binding protein  **CREB** .  Ref: 12933667 Endocrinology, 2003
  - We have demonstrated that  **CREB**  phosphorylation in the olfactory bulb OB is important for aversive olfactory learning in young rats, yet whether MAPK  **ERK**  functions as an upstream regulator are necessary for this olfactory learning remains to be determined.  Ref: 12946695 Neuroscience, 2003
  - Helicobacter pylori stimulates host cyclooxygenase 2 gene transcription critical importance of MEK  **ERK**  dependent activation of USF1 2 and  **CREB**  transcription factors.  Ref: 14531897 Cell Microbiol, 2003
  - On the other hand, inhibition of a mitogen activated protein kinase MAPK extracellular signal regulated kinase  **ERK**  pathway by a MAPK  **ERK**  kinase MEK inhibitor PD98059 suppressed both neuritogenesis and neurite outgrowth without  **CREB**  phosphorylation.  Ref: 14648587 J Neurosci Res, 2003
  - Both  **ERK**  and  **CREB**  were rephosphorylated by a second period of depolarization that followed a recovery period of 10 min or more.  Ref: 12832466 Mol Cell Biol, 2003
  - Both drugs increased the levels of activated phospho ERK44 42, activated phospho ribosomal protein S6 kinase 1 RSK1 a substrate of  **ERK** , phospho  **CREB**  cAMP response element binding protein and phospho B cell lymphoma protein 2 antagonist of cell death substrates of RSK, and BDNF.  Ref: 12917364 J Neurosci, 2003
  - PKC inhibition also impaired context and tone dependent fear conditioning and prevented the phosphorylation of  **Erk**  1 2, Elk 1, and  **CREB** , without affecting p90Rsk 1.  Ref: 14739006 Behav Brain Res, 2004
  - Incubation of unstimulated hippocampal slices with YC 1 plus nitroprusside increased the immunofluorescence of phospho extracellular signal regulated kinase  **ERK**  and phospho cAMP response element binding protein  **CREB** .  Ref: 12761342 Mol Pharmacol, 2003
  - Recently, it has been shown that  **ERK**  extracellular signal regulated kinase 1 2, protein kinase A PKA, and  **CREB**  cAMP response element binding protein are crucial factors for experience dependent development of the visual cortex, but very little is known about the role of visual experience in their activation.  Ref: 12904462 J Neurosci, 2003
  - Exogenous ET 1 mimicked nonmyocyte conditioned media mediated  **ERK**  and  **CREB**  phosphorylation and Bcl 2 protein increase but not Akt phosphorylation.  Ref: 14736733 EndocrinologyEndocrinology, 2003
  - We investigated the involvement of hippocampal protein kinase A PKA, protein kinase C PKC, calcium calmodulin dependent protein kinases CaMK II, and mitogen activated extracellular signal regulated kinases 1 and 2 Mek 1 2 in the phosphorylation of their downstream targets extracellular signal regulated kinases 1 and 2  **Erk**  1 2, cAMP response element binding protein  **CREB** , Elk 1, and p90 ribosomal S6 kinase 1 p90Rsk 1.  Ref: 14739006 Behav Brain Res, 2004
  - To analyze the downstream consequences of  **ERK**  and PKA signaling, we studied the action of visual stimulation on transcription of genes controlled by  **CREB**  in transgenic mice carrying the LacZ reporter gene under the control of the CRE cAMP response element promoter.  Ref: 12904462 J Neurosci, 2003
  - The current study is to investigate whether NAMDA induced neuroprotection occurs via the activation of  **ERK**  and its downstream effector,  **CREB** .  Ref: 14667449 Neuroscience, 2004
  - Taken together with the previous findings, these results indicate that the MAPK  **ERK**   **CREB**  pathway is required for the long term... facilitation process of aversive olfactory learning in young rats.  Ref: 12946695 Neuroscience, 2003
  - Moreover, blockade of NMDA receptor NMDAR did not decrease FAK, MAPK  **ERK** , and  **CREB**  phosphorylation although LTP induction was blunted by NMDAR antagonist.  Ref: 12764094 J Neurosci, 2003
  - However, a role of the mitogen activated protein kinase extracellular signal regulated kinase kinase MAPK  **ERK**  or MEK, an upstream kinase for  **CREB** , and its relation to  **CREB**  phosphorylation in neuroprotection in cerebral ischemia has not been delineated.  Ref: 14667449 Neuroscience, 2004
  - Finally, immunostaining for the phosphorylated form of cAMP response element binding  **CREB**  protein, a downstream target of the  **ERK**  MAPK cascade, was increased in perilesional glia 7 d after FSL.  Ref: 12866812 J Neurotrauma, 2003
  - Removal of magnesium also resulted in induction of the MAPK  **ERK**  substrate mitogen and stress response kinase 1 MSK1 and induced phosphorylation of the MSK1 substrate, the transcription factor cAMP response element binding protein  **CREB** .  Ref: 12807421 J Neurochem< MedlineTA>J Neurochem, 2003
  - Inhibition of PKA impaired context and tone dependent fear conditioning and significantly reduced the phosphorylation of Elk 1, p90Rsk 1,  **Erk**  1 2, and  **CREB** .  Ref: 14739006 Behav Brain Res, 2004
  - The effects of the durations of depolarization and interpulse recovery on reactivation of  **ERK**  and  **CREB**  were characterized.  Ref: 12832466 Mol Cell Biol, 2003
  - Levels of the prylated, active forms of MEK,  **ERK**  1 2, RSK1 and  **CREB** , and the expression levels of Bcl 2 family were assayed by Western Blotting.  Ref: 14653110 Hunan Yi Ke Da Xue Xue Bao, 2003
  - TRAIL induced distinct short term 1 60 min and long term 3 24 h effects on the protein kinase B PKB Akt Akt, extracellular signal regulated kinase  **ERK** , cAMP response element binding protein  **CREB** , nuclear factor kappa B NF kappaB and caspase pathways.  Ref: 12807432 J Neurochem< MedlineTA>J Neurochem, 2003
  - MAPK  **ERK**  and  **CREB**  are the downstream events of FAK activation.  Ref: 12764094 J Neurosci, 2003
  - This opposite regulation was specifically exerted by haloperidol and clozapine on  **ERK** ,  **CREB** , and Elk 1 phosphorylation, as both anti psychotic drugs increased the phosphorylation of the dopamine and cyclic AMP regulated phosphoprotein of 32 kDa DARPP 32 at the cyclic AMP dependent protein kinase PKA site.  Ref: 12871586 J Neurochem, 2003
  - Similarly, animals treated with NAMDA following ischemia showed increased  **ERK**  and  **CREB**  phosphorylation in the CA1 subregion of the hippocampus during early reperfusion period with increased number of surviving neurons examined 7 days following ischemia.  Ref: 14667449 Neuroscience, 2004
  - The results showed that the  **ERK**   **CREB**  signaling pathway might be involved in NAMDA induced neuroprotection following transient global ischemia and imply that the activation of the pathway in neurons may be an effective therapeutic strategy to treat stroke or other neurological syndromes.  Ref: 14667449 Neuroscience, 2004
  - In addition, ET A receptor antagonists BQ123 and BQ485 partially blocked nonmyocyte conditioned media mediated antiapoptotic effect,  **ERK**  and  **CREB**  phosphorylation, and Bcl 2 protein increase.  Ref: 14736733 EndocrinologyEndocrinology, 2004
  - **Erk**  1 2, Elk 1, and  **CREB**  were identified as common targets of PKA, PKC, and CaMK II during memory consolidation the time window and sequence of their phosphorylation was specific for the individual kinase.  Ref: 14739006 Behav Brain Res, 2004
  - Inhibition of CaMK II did not affect fear conditioning and reduced the phosphorylation of  **Erk**  1 2, Elk 1,  **CREB** , and p90Rsk 1 only transiently.  Ref: 14739006 Behav Brain Res, 2004
  - Lithium increased the activities of MEK,  **ERK**  1 2, RSK1, and  **CREB** , up regulated the expression of Bcl 2, and down regulated the expression of Bax in rat brain hippocampus and frontal cortex.  Ref: 14653110 Hunan Yi Ke Da Xue Xue Bao, 2003
